# Conveyor Belt Deviation Detection for Mineral Mining Applications Based on Attention Mechanism and Boundary Constraints

**DOI:** 10.3390/s25226945

**Published:** 2025-11-13

**Authors:** Long Ma, Jiaming Han, Chong Dong, Ting Fang, Wensheng Liu, Xianhua He

**Affiliations:** 1 School of Electrical and Information Engineering, Anhui University of Technology, Ma’anshan 243002, China; malong@ahut.edu.cn (L.M.); rjdongchong@163.com (C.D.); flyting_69@163.com (T.F.); 2 Anhui Masteel Mining Resources Group Co., Ltd., Ma’anshan 243071, China; 18455595206@163.com (W.L.); 13855512642@163.com (X.H.)

**Keywords:** conveyor belt deviation, deep learning, semantic segmentation, attention mechanism

## Abstract

To address the issue of material spillage and equipment wear caused by conveyor belt deviation in complex industrial scenarios, this study proposes a detection method based on an improved U-Net. The approach adopts U-Net as the backbone network, with a ResNet34 encoder to enhance feature extraction capability. At the skip connections, a Multi-scale Adaptive Guidance Attention (MASAG) module is embedded to strengthen the fusion of semantic and detailed features. In the loss function design, a boundary loss is incorporated to improve edge segmentation accuracy. Furthermore, the segmentation results are refined via edge detection and RANSAC regression, and a reference line is constructed based on the physical stability of rollers in the image to enable quantitative measurement of deviation. Experiments on a self-constructed dataset demonstrate that the proposed method achieves higher accuracy (99.77%) compared with the baseline U-Net (99.65%) and also surpasses other categories of approaches, including detection-based (YOLOv5s), anchor-point-based (UFLD), and segmentation-based approaches represented by SEU-Net and DeepLabV3+, thereby exhibiting strong robustness and real-time performance across diverse complex operating conditions. The results validate the effectiveness of this method in practical applications and provide a reliable technical pathway for the development of intelligent monitoring systems for mining conveyor belts.

## 1. Introduction

**Figure 1:**
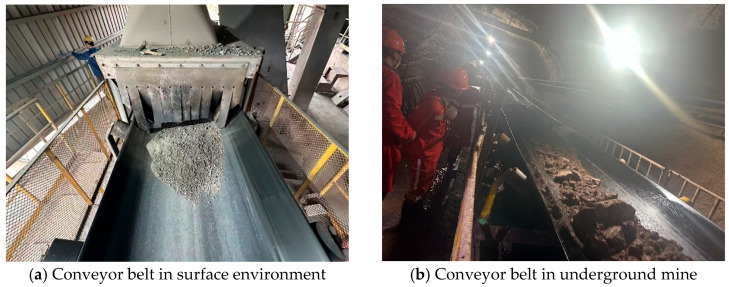
Representative applications of conveyor belts in different operational scenarios.

A mining conveyor belt is a type of equipment that employs a flexible belt body as the load-bearing component, while material transportation is achieved through the interaction of drive pulleys and idler rollers. The primary function of the conveyor belt lies in its ability to efficiently and continuously carry and transport bulk materials or unit goods, thereby meeting the demands of large-scale material handling in mining, metallurgy, and port operations [1,2]. Depending on the application scenario, conveyor belts can be extensively deployed in both surface environments and underground mines. A typical application is illustrated in Figure 1.

During long-term operation, conveyor belt systems frequently experience belt deviation, which refers to the lateral displacement of the belt from its designated central trajectory during motion. This phenomenon is highly common in continuous transportation systems such as those in mining, metallurgy, and port industries. If not detected and corrected in time, even minor deviations can lead to material spillage and equipment wear, while severe cases may cause belt tearing, roller-frame damage, or other safety hazards that threaten the continuity and stability of production. Belt deviation usually results from a combination of mechanical and operational factors. The main causes include inaccurate positioning of idlers and pulleys during installation, frame misalignment, improper tension adjustment leading to uneven stress distribution, poor joint quality that introduces thickness or stiffness inconsistencies, uneven material loading that causes lateral bias, and belt aging or surface wear during prolonged service. These factors collectively cause the belt to gradually deviate from its centerline, producing either continuous or intermittent offset behavior. Therefore, real-time monitoring and automated identification of conveyor belt deviation are of great engineering significance for ensuring the safe and stable operation of conveying systems and reducing maintenance costs [3].

The phenomenon of conveyor belt deviation not only disrupts the normal operation of conveying systems but also leads to a series of serious consequences. Deviation causes continuous friction between the belt edge and structural components such as the frame and idlers, resulting in abnormal local wear and surface peeling of the belt. In severe cases, this can lead to belt tearing or breakage, significantly reducing the service life and operational reliability of the conveyor system. Moreover, serious deviation often causes material spillage, which increases cleaning and maintenance workloads and may even block sensors or motor inlets, further affecting the continuity of system operation. When the deviation becomes excessive, the conveyor must be shut down for adjustment, leading to production interruptions, economic losses, and potential safety risks for both equipment and personnel. Consequently, conveyor belt deviation has become one of the major hidden hazards affecting the stable operation of conveying systems in industries such as mining and metallurgy [4].

**Figure 2:**
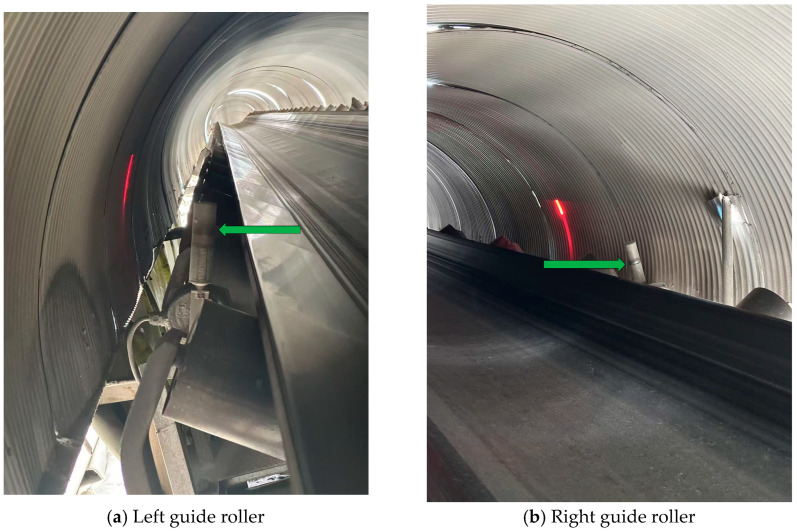
The arrows in (**a**,**b**) indicate the mechanical anti-deviation guide rollers located on the left and right sides of the conveyor belt, respectively.

Traditionally, mechanical anti-deviation devices have been employed to address the problem of conveyor belt deviation. Vertical rollers are installed on both sides of the belt, and when deviation occurs, the belt comes into contact with the rollers and is subjected to a lateral force that forces it back to the normal position [5]. Such devices provide correction and protection when severe deviations occur. However, they are only activated once the belt has deviated beyond a certain threshold, can counteract only part of the displacement, and are incapable of quantifying the real-time operational status of the conveyor belt. Moreover, prolonged friction between the belt and the vertical rollers leads to edge wear, thereby shortening the service life of the belt. Consequently, mechanical anti-deviation devices fall short of meeting the requirements for real-time monitoring and reliable detection of belt deviation. An example of their application in an operational conveyor belt system is illustrated in the figure below. Figure 2 illustrates the mechanical anti-deviation guide rollers positioned on both sides of the conveyor belt, as indicated by the arrows in (a) and (b).

Machine vision, as an important branch of artificial intelligence and computer vision, utilizes computers and image processing algorithms to simulate human visual perception, extracting essential information from images or videos to accomplish tasks such as recognition, detection, and measurement. In recent years, with the rapid advancement of deep learning and computing power, machine vision has gradually replaced traditional manual inspection and, in certain cases, mechanical sensor-based detection, becoming a key enabling technology for industrial automation and intelligent manufacturing.

The development of machine vision is closely tied to the continuous evolution of image recognition algorithms. The rise of deep learning in the field of computer vision has driven the rapid advancement of convolutional neural network (CNN) algorithms over the past decade. Numerous researchers have proposed a wide range of efficient models for tasks such as object detection, object classification, and semantic segmentation, thereby accelerating technological progress. The iterative improvement of these algorithms has not only enhanced the accuracy and efficiency of image recognition but also opened new avenues for conveyor belt deviation detection in industrial settings.

Object detection aims to determine the presence of objects within an image and to provide their locations and bounding boxes. Early algorithms, such as R-CNN and Faster R-CNN, established the two-stage framework based on region proposals [6]. Single-stage methods, including the YOLO series and SSD, introduced an end-to-end approach, significantly improving detection speed [7,8]. In recent years, both the YOLO family and Transformer-based methods such as DETR have further achieved a balance between speed and accuracy [9]. In the context of conveyor belt deviation detection, some studies have attempted to use object detection techniques to extract belt edge regions, estimating the edges through the diagonals of rectangular bounding boxes. However, this approach is highly dependent on boundary precision and exhibits limited robustness in complex operational environments.

Object classification aims to determine the category of an image based on learned feature patterns. Networks such as AlexNet, VGG, ResNet, DenseNet, and EfficientNet have progressively improved classification performance, serving as important backbones for subsequent tasks [10,11,12,13,14]. More recently, the emergence of Vision Transformer (ViT) has further advanced large-scale image classification [15]. In the context of conveyor belt deviation detection, classification-based methods are typically employed to determine whether a belt is in a misaligned state. While these methods are straightforward to implement and computationally efficient, they can only provide the overall status of the conveyor belt and cannot specify the degree of deviation or the precise location of deviation, thereby falling short of the spatial information requirements in real-world operational scenarios.

Semantic segmentation involves assigning a semantic category to each pixel in an image, thereby providing a detailed representation of target regions. Compared with object detection and classification tasks, semantic segmentation offers a higher level of spatial information. It has been widely applied in fields such as medical imaging, autonomous driving, and industrial inspection. The Fully Convolutional Network (FCN) first introduced an end-to-end framework for pixel-level prediction, enabling segmentation tasks to be optimized using deep convolutional neural networks [16]. Architectures such as U-Net and SegNet leverage symmetric encoder–decoder structures and multi-scale feature fusion to further enhance segmentation performance, particularly for small targets and complex backgrounds [17,18]. More recently, the DeepLab series has employed atrous convolutions and conditional random fields to model multi-scale contextual information [19], HRNet maintains high-resolution features to enhance edge detail representation [20], and Transformer-based methods such as SegFormer and Mask2Former combine global modeling with multi-scale feature representation [21,22], achieving a new balance between accuracy and computational efficiency. In conveyor belt deviation detection, semantic segmentation offers distinct advantages: by performing pixel-wise recognition of the entire belt region, it enables precise extraction of the left and right edges, facilitating accurate delineation of the conveyor belt boundaries.

In summary, traditional mechanical anti-deviation devices exhibit notable limitations in terms of real-time performance and reliability. Although existing machine vision methods have achieved significant progress in various aspects, their applicability to conveyor belt deviation detection remains constrained. To address these challenges, this study aims to achieve precise detection and reliable recognition of conveyor belt deviation, building upon existing research. The main contributions of this work are as follows:

(1) Based on the existing Multi-scale Adaptive Guidance Attention (MASAG) mechanism, the structure is adapted to the conveyor belt deviation detection task and embedded into the skip connections of U-Net. This integration enhances the cross-scale fusion of semantic and edge features, thereby improving the model’s capability for accurate edge recognition under complex working conditions. Compared with SEU-Net, the proposed design achieves more effective boundary feature enhancement and yields superior edge segmentation performance.
(2) A belt center localization method based on the physical stability of idler edges is developed, allowing for the quantitative analysis of deviation severity.
(3) A cross-method experimental validation strategy is employed: U-Net is used as the baseline model to verify improvements in segmentation accuracy, and comparative experiments are conducted with the object detection model YOLOv5s and the lane detection model UFLD [23], evaluating performance differences among different methodological approaches.
(4) A comprehensive dataset was constructed, covering multiple environmental conditions such as normal illumination, strong light, low light, and rainy weather. The dataset provides a reliable foundation for model training and performance evaluation under diverse real-world scenarios.

## 2. Materials and Methods

Semantic segmentation methods provide dense, pixel-level predictions that enable the extraction of continuous and detailed belt contours, offering a more interpretable and geometrically consistent foundation for deviation computation. This pixel-wise modeling capability makes semantic segmentation particularly suitable for handling complex backgrounds and lighting variations commonly present in mining environments.

To achieve precise recognition of potential conveyor belt edges, this study proposes an improved U-Net architecture to address the insufficient edge-feature extraction capability of existing semantic segmentation models under complex environmental conditions [24].

Compared with high-complexity models such as DeepLab, FPN, and Vision Transformer, U-Net features a relatively simple structure with strong customizability and scalability, allowing flexible adaptation to specific industrial scenarios. Therefore, U-Net is selected as the baseline framework of this study, upon which structural optimizations are performed for the conveyor belt deviation detection task.

The proposed improvement integrates a ResNet encoder and a Multi-scale Adaptive Guidance Attention (MASAG) mechanism [25].

In this design, the ResNet encoder preserves low-level spatial features essential for edge recognition through residual connections, while the MASAG module adaptively fuses multi-scale contextual information to enhance the model’s ability to detect potential edges under complex operating conditions, thereby improving the accuracy and robustness of deviation detection.

After obtaining the binarized segmentation of the conveyor belt region, the edges are preliminarily visible but often exhibit pixel-level jaggedness or noise, resulting in insufficient geometric accuracy for potential edge localization. To address this, the Canny edge detection algorithm is introduced to post-process the segmentation results. The Canny algorithm, with its noise robustness and single-pixel edge localization precision, can extract continuous and smooth structural contours from the binarized regions produced by the segmentation method [26]. This step effectively mitigates the problem of coarse edges in semantic segmentation outputs and provides an accurate set of edge points for subsequent line fitting.

**Figure 3:**
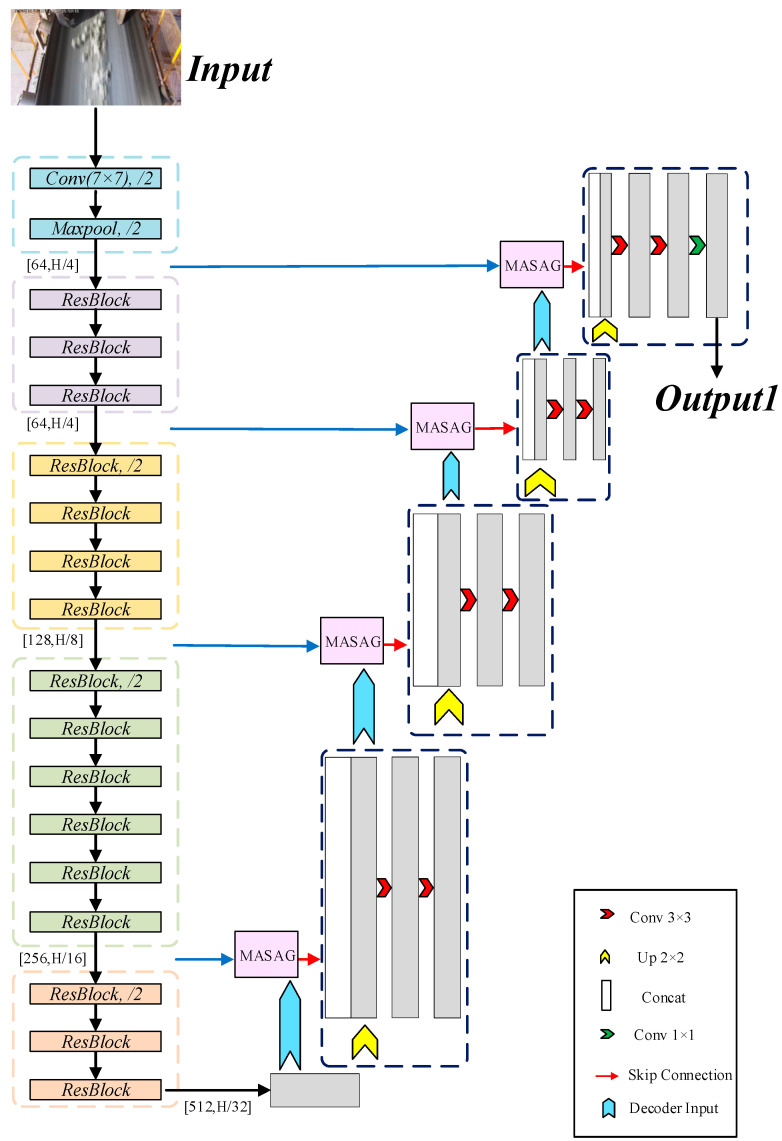
Architecture of the improved semantic segmentation network.

After obtaining the edge point set, the RANSAC regression algorithm is employed for line fitting. By iteratively performing random sampling [27], RANSAC can effectively eliminate outliers caused by residual noise or local defects, allowing for the estimation of optimal linear models for the edges on both sides of the conveyor belt and achieving precise edge localization. Ultimately, by calculating the pixel distance between the fitted lines and a predefined central reference line, the degree of conveyor belt deviation can be accurately measured, enabling automated deviation detection. Figure 3 illustrates the architecture of the improved U-Net network.

### 2.1. U-Net Network

U-Net features a classical symmetric encoder–decoder architecture that has become a foundational paradigm for semantic segmentation. It continues to serve as a core backbone in numerous state-of-the-art studies and has been widely adopted and extended. The model extracts hierarchical semantic features through the encoder path via downsampling, while the decoder path performs upsampling to recover spatial details. Its pioneering skip connection mechanism enables the fusion of multi-scale features from the encoder with corresponding stages in the decoder, effectively mitigating the loss of fine-grained information in deep networks.

**Figure 4:**
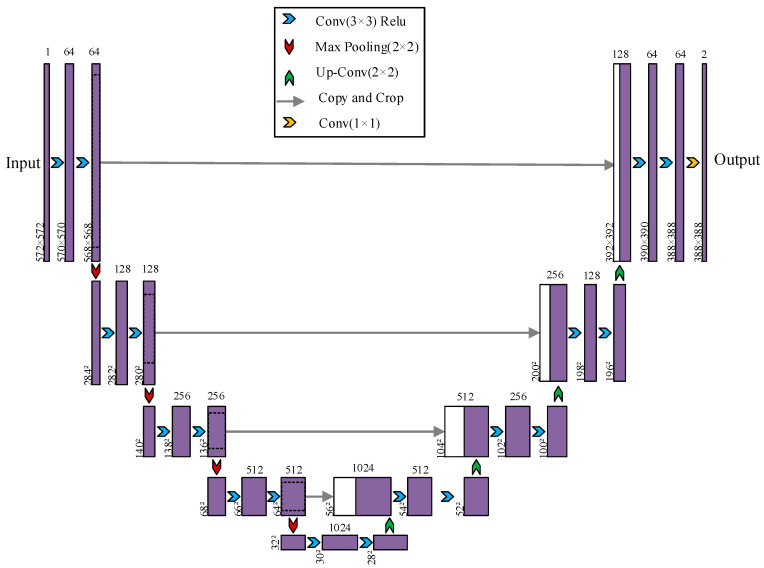
U-Net network model.

Although U-Net was originally developed for biomedical image analysis, its modular design endows it with strong extensibility. The conveyor belt edge recognition task addressed in this study requires capturing subtle and highly variable edge features from a large volume of industrial images. Therefore, U-Net is employed as the baseline model, allowing the network to achieve powerful semantic representation while producing high-precision segmentation contours rich in detail. The U-Net network model is illustrated in Figure 4.

However, the original U-Net model exhibits limitations in conveyor belt region recognition tasks under complex industrial environments. Its encoder has restricted feature extraction capability, and the model shows insufficient adaptability to varying illumination and complex backgrounds, which constrains further improvements in segmentation accuracy. To address these issues, two modifications are introduced based on the U-Net baseline. First, the original encoder is replaced with a ResNet residual network to enhance feature extraction capability and mitigate network degradation. Second, a multi-scale attention gating module is integrated to improve the model’s robustness under complex conditions, thereby enabling more accurate and stable segmentation of conveyor belt regions.

### 2.2. Improved U-Net Model

To enhance the model’s ability to extract potential conveyor belt edges under complex operational conditions, the original U-Net encoder is replaced with a ResNet-34 network [28]. ResNet, through its core residual learning mechanism, addresses the problems of gradient vanishing and network degradation in deep network training.

**Figure 5:**
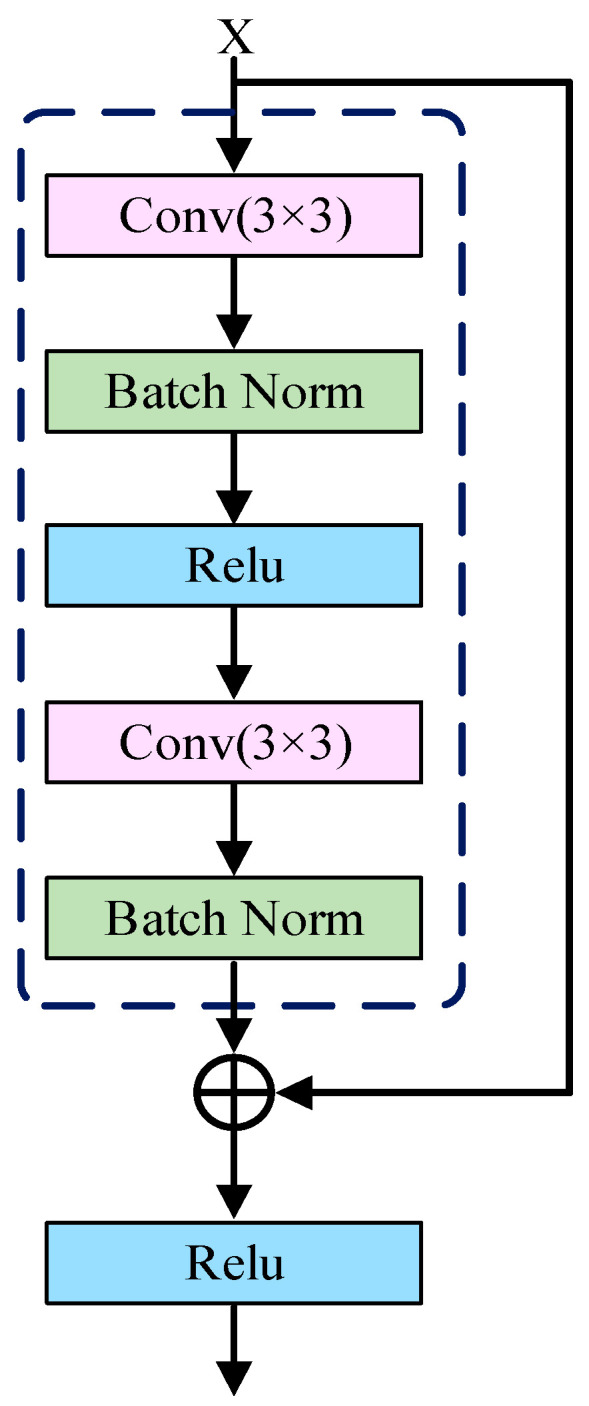
Structure of the residual block.

The fundamental principle of ResNet is the introduction of residual blocks with shortcut connections, which allow the network to learn the residual mapping between inputs and outputs. The input features are transformed through a series of convolutional layers along the main path, while an identity mapping preserves the original input along a shortcut path. The outputs from both paths are then combined via element-wise addition. This structure effectively mitigates the gradient vanishing problem in U-Net, facilitates the learning of identity mappings, and allows the network to increase in depth without performance degradation, while efficiently preserving and integrating low-level detail features with high-level semantic representations. The structure of the residual block is illustrated in Figure 5.

**Figure 6:**
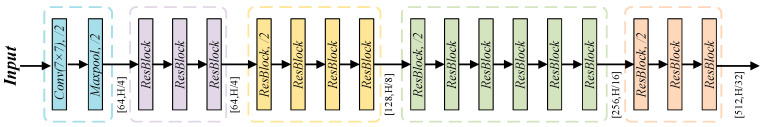
ResNet34 residual structure.

To integrate the ResNet network as the feature extraction backbone with the U-Net decoder, the final average pooling and fully connected layers of ResNet are removed. The structure of the ResNet module used in this study is illustrated in Figure 6.

However, conveyor belt region recognition presents an inherent challenge: the idlers supporting the belt exhibit similar color and texture characteristics to the belt itself. Consequently, both the original U-Net encoder and the ResNet residual structure capture not only the true conveyor belt edges but also interfering idler information. In the original U-Net, the skip connection mechanism concatenates all encoder features directly with the decoder, which makes it difficult to effectively distinguish between such similar features. Replacing the encoder with a more expressive ResNet enhances high-level semantic features, but the shallow features transmitted from the encoder also become richer and more complex, thereby exacerbating the limitations of the original skip connections in feature selection. As a result, the model cannot adaptively focus on the conveyor belt itself, causing the decoder to struggle in reconstructing details accurately; this may lead to erroneous segmentation boundaries or inclusion of adjacent idlers within the segmented belt region. Therefore, a fusion strategy capable of actively guiding the model to focus on the target while suppressing similar interferences is required to replace simple concatenation.

**Figure 7:**
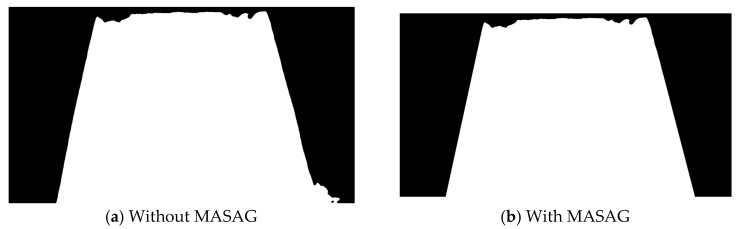
Panel (**a**) shows the segmented conveyor belt region without the MASAG module, where the right side includes the binarized idler area. Panel (**b**) shows the segmented conveyor belt region with the MASAG module, effectively mitigating the influence of idlers on the edge detection.

To address the issues arising from both the inherent task challenge and the limitations of the original skip connections, a MASAG module is introduced into the skip connections. This module takes as input the multi-scale low-level features from the encoder and the high-level semantic features from the current decoder stage, and computes adaptive weights to generate an attention map that dynamically focuses on key regions. This approach suppresses feature responses associated with background interferences such as idlers while actively enhancing the representation of potential conveyor belt edges. The MASAG module guides task-relevant regions into the decoder, directly mitigating interference from similar objects and ultimately improving segmentation accuracy and boundary clarity. The structure of the MASAG module used in this study is illustrated in the figure below, with a comparison of results before and after the introduction of this attention mechanism shown in Figure 7.

**Figure 8:**
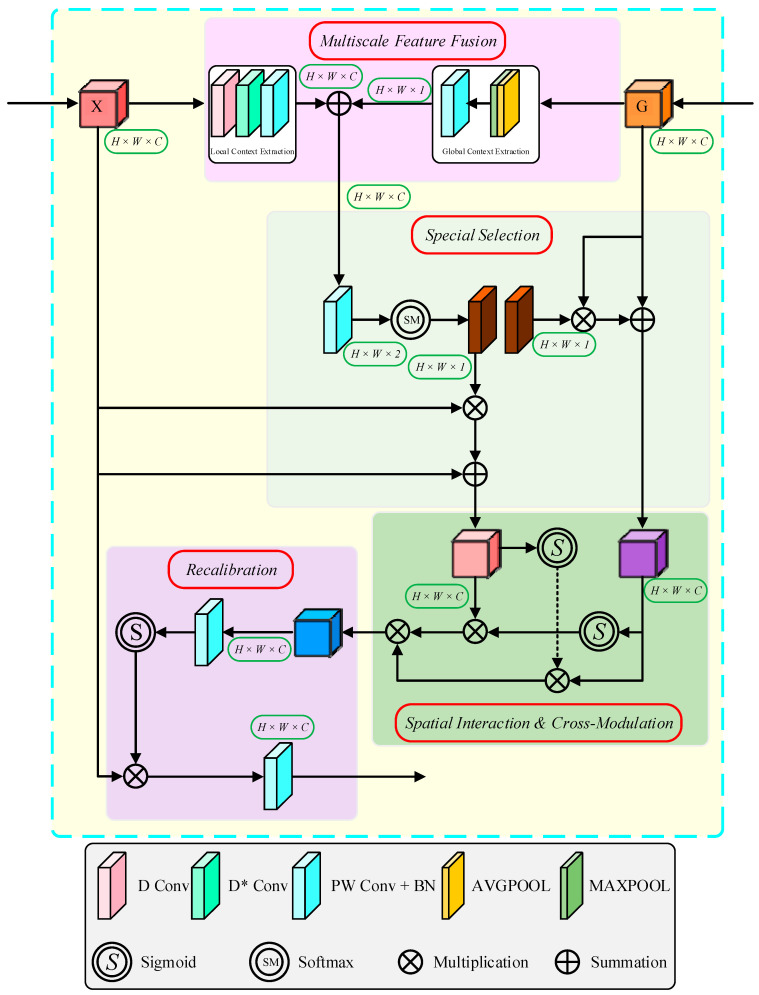
MASAG Module.

As shown in Figure 8, the MASAG module receives high-resolution detail features (X) from the ResNet encoder and high-level semantic features (G) from the U-Net decoder.

The information processing in MASAG mainly consists of four stages: multi-scale fusion, spatial selection, spatial interaction with cross-modulation, and recalibration.

In the multi-scale fusion stage, the encoder features X undergo local context extraction. The input features are first processed through a depth-wise convolution to capture high-resolution local spatial information and fine-grained details. This is followed by a dilated convolution to further capture high-resolution local spatial information and detail features. Finally, a 1 × 1 convolution is applied to obtain the fused local context features.

For the decoder guidance features G global context modeling is performed. A dual-path pooling strategy is employed, consisting of global average pooling and global max pooling on G, aggregating semantic information from different perspectives. The resulting feature vectors are concatenated along the channel dimension and fused and compressed through a 1 × 1 convolution. This stage enhances the segmentation accuracy and boundary clarity of the conveyor belt region.

The multi-scale feature fusion can be expressed mathematically as follows:

(1) X=C(D*(D(X)))

(2) $$G=C_{g}(Concat[AVGPOOL(G),MAXPOOL(G)])$$

(3) F=X+G

In the above equations, D(⋅) denotes a depth-wise convolution, $D^{*}(\cdot )$ denotes a dilated convolution, and C(⋅) denotes a 1 × 1 convolution. AVGPOOL and MAXPOOL represent global average pooling and global max pooling, respectively, while Concat[⋅] denotes channel-wise concatenation. $C_{g}(\cdot )$ represents the 1 × 1 convolution fusion.

During the spatial selection phase, the feature F from Equation (3) is projected onto dual-channel feature maps to align it with the input features X and G. A Softmax function is applied along the channel dimension to compute the spatial selection weights [29], which are used to finely adjust the principal stress distributions of X and G. This process yields spatially selected features $X_{1}$ and $G_{1}$, helping to reduce interference from irrelevant background regions in conveyor belt scenarios. The procedure is mathematically formulated as follows:

(4) M=C(F)

(5) $$\left[W_{1},W_{2}]=Soft\max(M,\dim=1\right)$$

This operation ensures that at each spatial position (h,w), the sum of the two-channel weights remains equal to 1. The weight maps $W_{1}$ and $W_{2}$ quantitatively represent the contributions from the encoder detail feature X and the decoder feature G, respectively, with their specific values being adaptively determined based on the local content at each position.

(6) $$X_{1}=X+(W_{1}⊗X)$$

(7) $$G_{1}=G+(W_{2}⊗G)$$

Among these, ⊗ denotes element-wise multiplication. The weights are used to modulate the original input features, thereby generating spatially selected versions of the features, denoted as $X_{1}$ and $G_{1}$.

In the spatial interaction and cross-modulation stage, the feature $X_{1}$, which contains rich fine-grained local details, is gated via a Sigmoid activation mechanism [30] and modulated by $G_{1}$ that incorporates weighted global contextual information. This process generates an enhanced feature $X_{2}$, where detailed local information and global semantics are effectively fused. The computational procedure can be expressed as follows:

(8) $$X_{2}=\sigma (G_{1})⊗X_{1}$$

Among these, σ(⋅) denotes the Sigmoid activation function. The purpose of this operation is to modulate each local detail feature in $X_{1}$ with the corresponding global contextual importance from $G_{1}$.

Meanwhile, the feature $G_{1}$, which encapsulates weighted global contextual information, incorporates the fine-grained local context from $X_{1}$ through a similar mechanism, thereby evolving into an enhanced feature $G_{2}$:

(9) $$G_{2}=\sigma (X_{1})⊗G_{1}$$

Finally, $X_{2}$ and $G_{2}$ are fused to generate cross-enhanced features, ensuring the model accurately segments both the conveyor belt regions and their boundaries.

(10) $$F_{1}=X_{2}⊗G_{2}$$

In the recalibration stage, the module performs refined processing on the output of Equation (10) to generate the final optimized feature output. Specifically, $F_{1}$ is first processed by a convolutional layer, followed by a Sigmoid activation function σ, to produce an attention map A. This attention map is then used to recalibrate the original input feature X from the encoder. By performing element-wise multiplication between X and A, spatial weighting is achieved for the feature representation. The computational procedure is formulated as follows:

(11) $$A=\sigma (C(F_{1})⊗X)$$

This process effectively enhances feature responses in conveyor belt regions while suppressing irrelevant background information. The weighted features are then projected and refined through convolution after integrating the information and adjusting the channel dimensions, ultimately yielding the final output X. The procedure is formulated as follows:

(12) $$X=C\left(A\right)$$

Through the series of transformations described above, the recalibration stage equips the encoder feature X with adaptive multi-scale receptive fields tailored by the preceding stages—multi-scale fusion, spatial selection, and interactive modulation. The final output feature X incorporates precise contextual awareness, enabling effective integration with features in the decoder component of the U-Net architecture. Thereby, it significantly enhances both the accuracy and robustness of conveyor belt region segmentation under complex environmental conditions.

### 2.3. Region–Boundary Joint Loss Function Design

In semantic segmentation tasks, the design of the loss function significantly influences model learning performance. For the specific application scenario of conveyor belt deviation addressed in this paper, we propose a region–boundary joint loss function to ensure segmentation accuracy at the regional level while further improving the precision of boundary contours.

The objective of the region loss function is to measure the discrepancy between the prediction and ground truth at the holistic region level. In this work, we adopt a combined loss consisting of Cross-Entropy loss and Dice loss [31,32].

For the conveyor belt deviation task, we formulate the problem as a binary segmentation task, distinguishing between the conveyor belt region and the background. To ensure accurate pixel-wise classification, Binary Cross-Entropy (BCE) loss is introduced under this binary setting. This loss measures the pixel-level differences between predicted probabilities and the ground truth. In the context of conveyor belt deviation segmentation, BCE effectively guides the model to distinguish the conveyor belt region from the background. However, due to the large proportion of the conveyor belt area in the image, relying solely on BCE tends to bias the model toward predicting the dominant large region, thereby insufficiently focusing on the edges. This can lead to blurred boundaries and adversely affect the subsequent accurate detection of belt deviation. The Binary Cross-Entropy loss is defined as follows:

(13) $$L_{CE}=-\frac{1}{N}\sum_{i=1}^{N}\left[y_{i}\log(p_{i})+(1-y_{i})\log(1-p_{i})\right]$$

Among these, N is the total number of pixels, $y_{i}\in \{0,1\}$ denotes the ground truth label, and $p_{i}\in [0,1]$ represents the predicted probability from the model.

To mitigate this limitation, we further introduce the Dice loss, which directly measures the overlap between the predicted region and the ground truth, offering inherent robustness to region size imbalance. The Dice loss optimizes the overall regional overlap ratio, enabling the model to maintain prediction accuracy for large areas while still accounting for precision in smaller regions. However, the Dice loss still provides insufficient constraint on boundaries, potentially resulting in blurred or jittery predicted contours. This is detrimental to the precise detection of conveyor belt deviation, which requires accurate edge information. The Dice loss is formulated as follows:

(14) $$L_{Dice}=1-\frac{2\sum_{i=1}^{N}p_{i}y_{i}+\epsilon }{\sum_{i=1}^{N}p_{i}+\sum_{i=1}^{N}y_{i}+\epsilon }$$

Among these, ϵ is a small constant introduced for numerical stability, approaching zero in value. In all experiments conducted in this study, ϵ is set to $10^{-6}$, thereby preventing division-by-zero errors when certain classes are entirely absent in the image or mask.

To address the boundary blurring issue, we employ a boundary loss based on the distance transform, which assigns higher weights to pixels closer to the true boundary. This approach directs the model to focus more intensively on the edge regions of the conveyor belt during training. Such a design is chosen to effectively improve edge prediction accuracy, thereby reducing errors in edge extraction for the precise quantification of conveyor belt deviation. Although the introduction of boundary loss increases computational overhead, its substantial advantage in improving edge precision is critical for the conveyor belt deviation detection task. The boundary loss is calculated as follows:

(15) $$L_{Boundary}=\frac{1}{N}\sum_{i=1}^{N}D(y_{i})•|y_{i}-p_{i}|$$

Among these, $D(y_{i})$ denotes the distance from pixel i to the nearest true boundary, thereby guiding the model to focus more on edge information during training.

In this work, we combine the Binary Cross-Entropy loss, Dice loss, and Boundary Loss through a weighted summation to form a region–boundary joint loss function:

(16) $$L_{RBCL}=\lambda_{1}(\alpha L_{CE}+(1-\alpha L_{Dice}))+\lambda_{2}L_{Boundary}$$

where α controls the weighting between the Cross-Entropy and Dice losses for regional optimization, while $\lambda_{1}$ and $\lambda_{2}$ regulate the contributions of the regional loss and boundary loss, respectively.

### 2.4. Precise Quantification Method for Conveyor Belt Deviation

In visual inspection systems for conveyor belt deviation, the installation position and angle of the camera significantly influence the establishment of the detection reference. In practical industrial settings, due to constraints such as equipment layout, spatial limitations, and safety regulations, cameras often cannot be installed at theoretically ideal positions, which may result in the optical axis not being strictly perpendicular to the conveyor belt plane. Such non-ideal installation conditions directly alter the geometric relationship between the conveyor belt and background structures in the image, introducing systematic errors into pixel distance-based deviation measurements.

**Figure 9:**
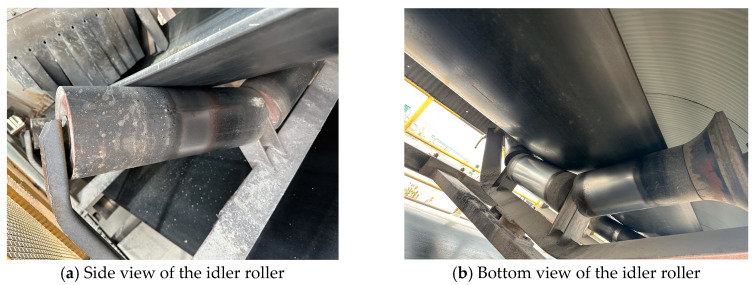
Images of the idler roller captured from different perspectives.

To address the above issue, this paper adopts a reference line construction method based on the edge positions of fixed idler rollers. The core advantage of this approach lies in shifting the measurement reference from the image boundaries, which depend on the camera coordinate system, to the idler rollers, which are physically fixed in position within the scene. As shown in Figure 9, images of the idler rollers were captured from different perspectives on site.

As a key load-bearing and guiding component of the conveyor belt system, the idler roller is manufactured from high-strength metal or composite materials, offering excellent mechanical stability and resistance to deformation. Mechanically, the idler roller is securely mounted onto the conveyor frame via rigid brackets, maintaining a constant spatial position and axial orientation during system operation. This material and structural invariance allow the edges of the idler roller to form a reliable spatial reference in the physical environment, unaffected by operational vibrations or long-term use.

By locating both edges of the idler roller and extending them to the image boundaries, a reference line is constructed by connecting the corresponding intersection points at the upper and lower image borders. Since this reference is derived directly from the inherent physical properties of the idler roller, it effectively mitigates deviation issues between the image coordinate system and the real-world coordinate system caused by non-ideal camera placement. This approach grounds the reliability of the reference construction on the fixed physical structures within the scene, rather than on the image coordinate system which is susceptible to installation variations, thereby enhancing the robustness and accuracy of deviation detection.

After obtaining the binary conveyor belt region output by the semantic segmentation model, the pixel-based area is converted into measurable geometric features to achieve precise quantification of the deviation magnitude. Edge detection is applied to the binary region to extract its contour as a continuous set of pixel points. A line fitting method is then used to derive the two edge lines representing both sides of the conveyor belt. The centerline between these two edge lines is calculated and compared pixel-wise with the reference line established based on the idler roller. This process transforms the belt’s deviation state into an accurate pixel-distance measurement.

#### 2.4.1. Reference Line Construction

**Figure 10:**
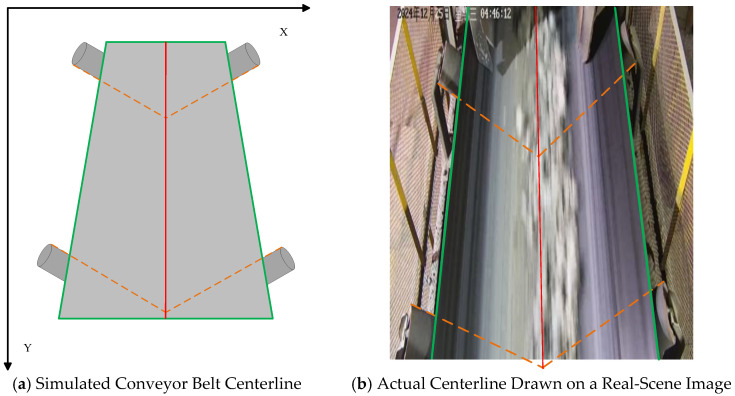
Illustration of two methods for drawing the conveyor belt centerline: (**a**) the simulated centerline constructed based on idler roller positions; (**b**) the actual centerline drawn on an in situ image.

The edge lines from both sides are extended to the upper and lower boundaries of the image, yielding two sets of intersection points. By connecting the corresponding upper and lower intersection points, a reference line for deviation measurement is constructed. This construction process is illustrated in Figure 10. The method for drawing the centerline is demonstrated below:

#### 2.4.2. Edge Extraction and Conveyor Belt Boundary Fitting

**Figure 11:**
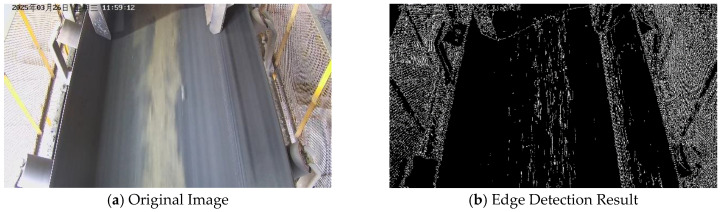
(**a**) Original image of the conveyor belt scene; (**b**) Result of applying edge detection directly to the original conveyor belt image.

This section takes the binary conveyor belt region output by the semantic segmentation module as input, and performs edge extraction and boundary line fitting based on this. The binary image output after semantic segmentation effectively isolates interference from complex backgrounds, providing a relatively clean conveyor belt region for edge detection. As shown in Figure 11, performing edge detection directly on the entire original image contains excessive complex information, making it difficult to accurately identify the conveyor belt edges.

The extracted binary conveyor belt region may exhibit ill-defined potential edges or locally irregular boundaries. Directly fitting lines to these potential edges could lead to inaccurate fitted lines for the conveyor belt, consequently compromising the accuracy of the deviation calculation. Therefore, an edge extraction and fitting method is further introduced based on the region obtained via semantic segmentation. The procedure is as follows:

(17) $$\begin{array}{l} G_{x}=\frac{\partial I}{\partial x}\\ G_{y}=\frac{\partial I}{\partial y}\\ G=\sqrt{{\left(G_{x}\right)}^{2}+{\left(G_{y}\right)}^{2}}\\ \theta =\arctan(\frac{G_{y}}{G_{x}}) \end{array}$$

where I(x,y) represents the input binary image.

After obtaining the gradient information, non-maximum suppression is applied to refine the edges to a single-pixel width. This is followed by a dual-threshold connection process (with lower threshold $T_{L}$ and higher threshold $T_{H}$). Although the input in our application scenario is already a binary conveyor belt region, the dual thresholds are utilized to assist in generating well-defined and continuous edges. The final output is an edge set E(x,y). The overall procedure is summarized as follows:

(18) $$E(x,y)=\left\{\begin{array}{l} 1,G(x,y)\geq T_{H}\\ 1,T_{L}\leq G(x,y)\leq T_{H}\\ 0,Others \end{array}\right.$$

**Figure 12:**
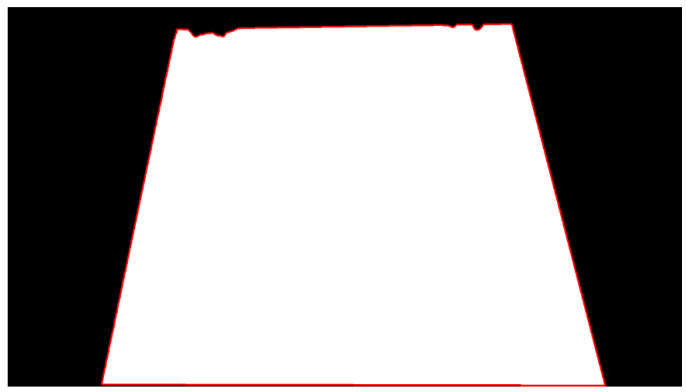
Binary image after edge detection.

As shown in Figure 12, the proposed method achieves more accurate edge localization.

After obtaining the edge-detected binary image, a row-wise scanning process is performed to record the leftmost and rightmost pixel positions in each row, forming two endpoint sets:

(19) $$LeftPoints=\{(y,x_{left}(y))|E(y,x_{left}(y))>0\}$$

(20) $$RightPoints=\{(y,x_{right}(y))|E(y,x_{right}(y))>0\}$$

At this stage, the edge image may still contain local noise and outliers. Direct fitting could result in lines that deviate from the actual conveyor belt boundaries. To address this, the collected sets of left and right edge points are, respectively, fitted using RANSAC regression to obtain robust and accurate boundary lines.

(21) $$\begin{array}{l} x_{left}(y)=a_{L}y+b_{L}\\ x_{right}(y)=a_{R}y+b_{R} \end{array}$$

The fitted lines are plotted on the image to visualize the conveyor belt boundary positions. Simultaneously, the centerline between the left and right edges is calculated to quantify the belt’s deviation status.

(22) $$x_{center}(y)=\frac{x_{left}(y)+x_{right}(y)}{2}$$

**Figure 13:**
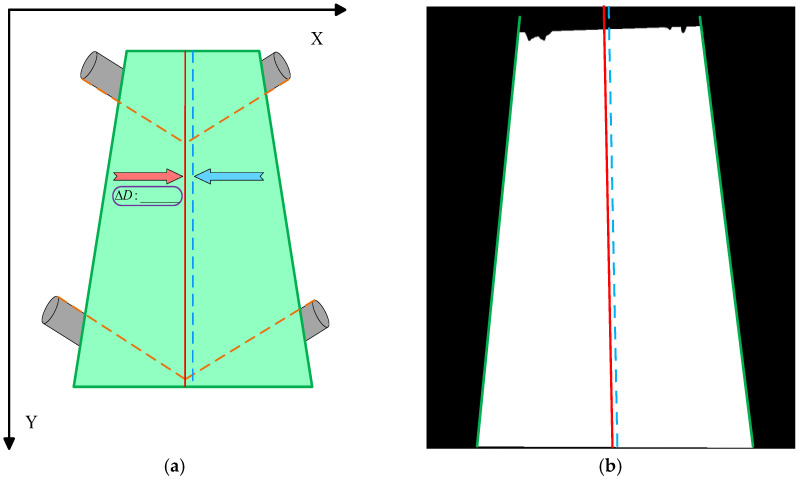
(**a**) Schematic illustration of deviation simulation in the conveyor belt region, (**b**) Schematic diagram of deviation detection in the binary image. (**a**) Demonstrates the extraction of actual conveyor belt edge lines through edge detection and Probabilistic Hough Transform line fitting, with the calculation of the displacement between the centerline and reference line for simulating deviation status; (**b**) Shows the algorithm’s detection results of the deviation state on the binary conveyor belt image.

As shown in Figure 13, the schematic diagram illustrates the quantification of conveyor belt deviation.

## 3. Results

### 3.1. Dataset Construction and Hardware Environment

**Figure 14:**
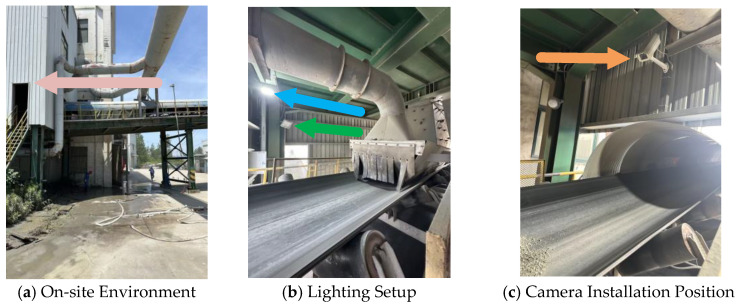
(**a**) The arrow indicates the installation scenario of the on-site camera; (**b**) The two arrows point to the supplemental lighting; (**c**) The arrow indicates the installation position of the on-site camera.

To validate the robustness of the proposed model in real industrial environments, this study adopts a case study approach, establishes a long-term, real-scene dataset collected from conveyor belt surfaces. The sample images are shown in Figure 14.

**Figure 15:**
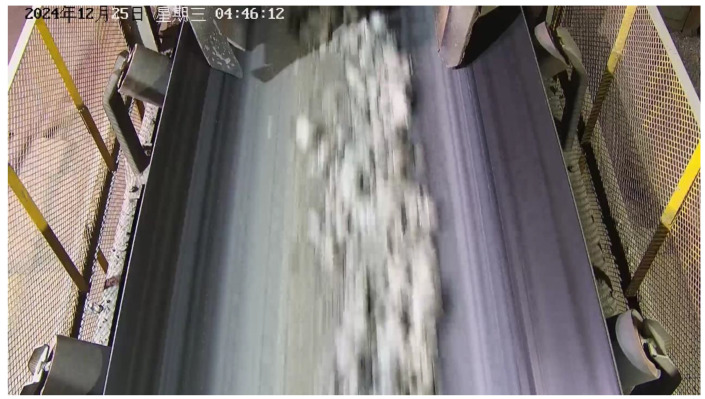
Normal Lighting Conditions.

**Figure 16:**
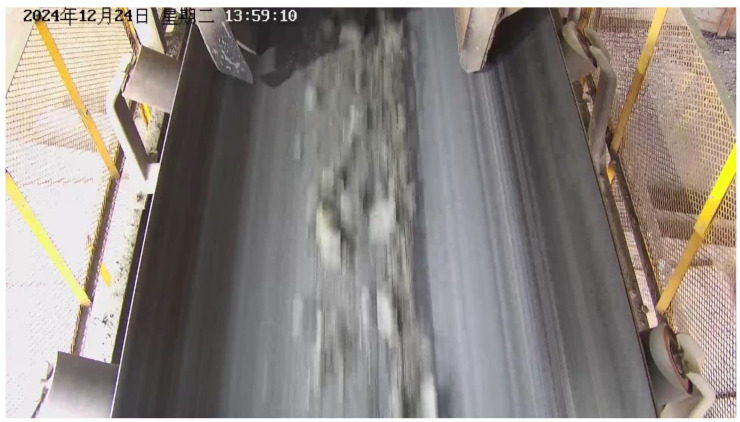
Strong Lighting Conditions.

**Figure 17:**
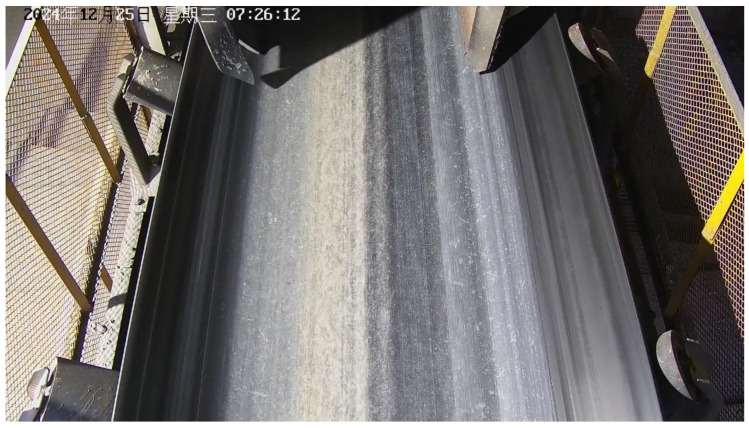
Low Lighting Conditions.

**Figure 18:**
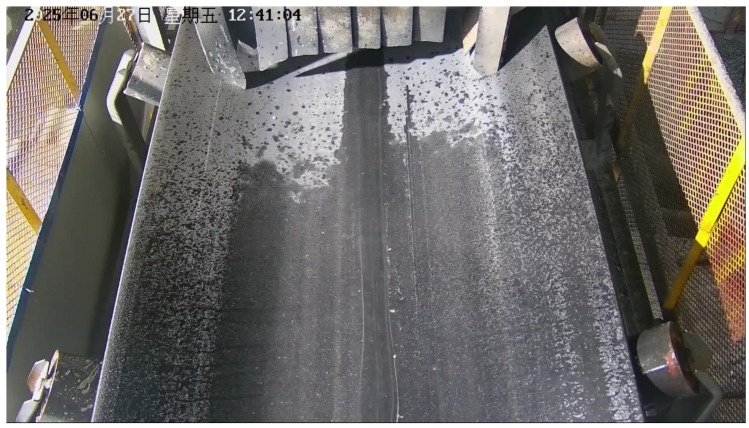
Rainy Conditions.

All data were collected from an actual mining site in Ma’anshan City, Anhui Province, with a collection period spanning six months. The acquisition was performed using fixed industrial cameras. The open structure on both sides of the collection site resulted in significant variations in internal lighting conditions, strongly influenced by time of day, weather, and seasonal changes, creating continuous variations from daytime brightness to nighttime darkness. This challenging lighting environment provides valuable test data for evaluating model performance under different illumination intensities, as demonstrated in Figure 15, Figure 16, Figure 17 and Figure 18.

**Figure 19:**
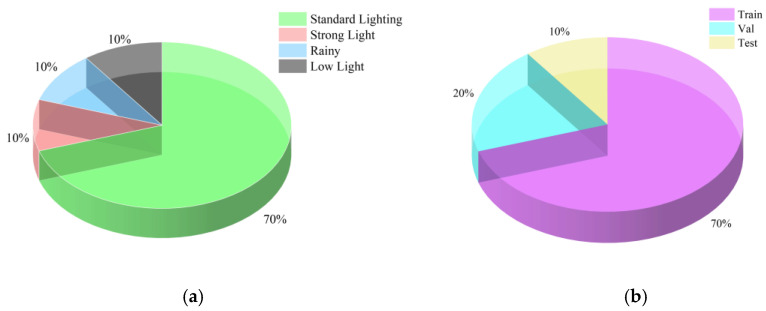
(**a**) Data Distribution Across Environmental Conditions, (**b**) Train/Validation/Test Split Within Each Environmental Category. Schematic diagram of data distribution.

All acquired original images underwent precise semantic segmentation to extract the conveyor belt regions. After screening, a curated selection of 10,000 representative images from the hundreds of thousands captured by the cameras was annotated. To rigorously evaluate the model’s generalization capability, the dataset was partitioned into training, validation, and test sets according to the actual environmental conditions. The value of this dataset lies in its authentic representation of the dynamic and challenging lighting variations present in real industrial settings. As shown in Figure 19, the specific distribution of the dataset is presented.

The on-site deployment environment is consistent with the experimental environment used in this study. The training parameters used in this study are summarized in Table 1 and Table 2.

### 3.2. Ablation Study

To verify the effectiveness of each proposed module, a series of systematic ablation experiments were conducted on the self-constructed dataset. The experimental settings and results are summarized in Table 3. The classical U-Net architecture was adopted as the baseline model, upon which ResNet34, the MASAG module, and the boundary loss function (Boundary Loss) were progressively integrated to evaluate the contribution of each component to model performance.

As shown in Table 3, the ablation experiments progressively validate the impact of each proposed component in this scenario. The results demonstrate that adopting ResNet34 as the encoder effectively improves performance, while the MASAG module further enhances feature representation through an attention mechanism. Finally, the introduction of the boundary loss function refines the segmentation accuracy along object boundaries. Overall, the proposed model achieves superior segmentation performance.

The loss curves during the training process are illustrated in the following figure.

**Figure 20:**
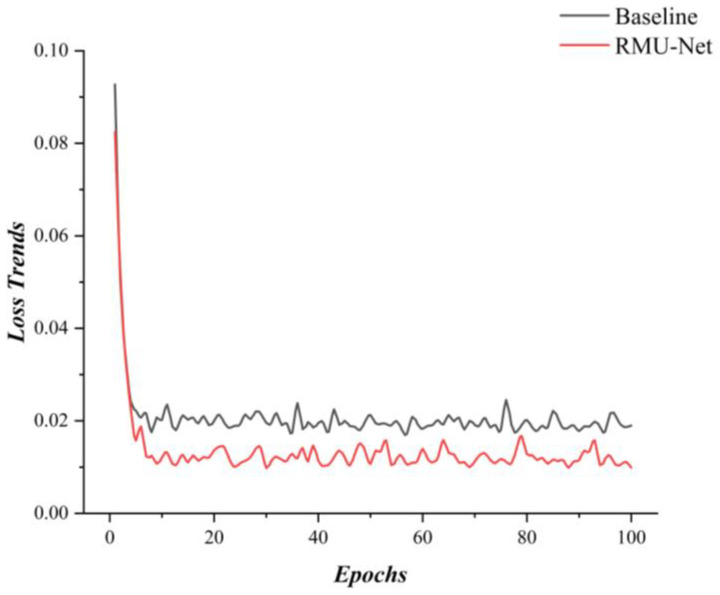
Loss curves of the Baseline U-Net and the improved RMU-Net during training.

Figure 20 presents a comparison between the U-Net model and the proposed ResNet + MASAG + Boundary configuration during the training process. All experimental data and conditions were kept identical.

### 3.3. Comparative Experiments

To comprehensively validate the effectiveness of the proposed model in the task of conveyor belt deviation detection, comparative experiments were conducted from multiple perspectives. At present, commonly used approaches can be broadly categorized into semantic segmentation-based methods and detection-based methods. Representative methods from both perspectives are introduced, with their principle illustrations and training curves provided, followed by comparative analysis against the proposed approach.

In the segmentation-based category, U-Net is adopted as the baseline model, while DeepLabV3+ and SEU-Net are used as comparative methods; in the object detection category, YOLOv5s is selected; and in the anchor-based detection category, UFLD is employed.

Table 4 summarizes the quantitative comparison of different methods in terms of accuracy, frame rate, and computational complexity (GFLOPs).

From the results presented in the table, it can be observed that segmentation-based methods demonstrate a significant advantage in pixel-level boundary localization, whereas object detection and anchor-based detection methods achieve higher frame rates but relatively lower accuracy.

In the context of this study, conveyor belt deviation detection prioritizes highly precise extraction of potential belt edges, as the quantitative measurement of deviation directly depends on the accuracy of edge localization. Even minor boundary errors can propagate through subsequent geometric fitting and quantitative deviation calculations, leading to cumulative errors that affect the reliability of early warnings. Therefore, although segmentation-based methods are slightly slower during inference, they offer irreplaceable advantages in terms of accuracy.

It should be noted that conveyor belt deviation represents a slowly developing anomaly, where displacement changes typically accumulate over seconds or even minutes. Hence, the frame rate requirement for such detection systems is relatively low. To balance real-time performance and accuracy, this study employs a frame-skipping detection approach, which significantly reduces computational load while maintaining sufficient temporal resolution to meet real-time monitoring needs.

To further verify the stability of the proposed method for conveyor belt deviation detection under different environmental conditions, the mean square error (MSE) between the detected centerline and the reference line was calculated.

All data were computed based on the testing set, covering four representative scenarios: normal illumination, strong-light interference, rainy conditions, and low-light environments.

This metric reflects the stability and accuracy of the algorithm in tracking the conveyor belt’s center position over time; a smaller MSE indicates that the detected trajectory is closer to the actual operating path and that the deviation measurement is more stable.

The results are summarized in Table 5.

**Figure 21:**
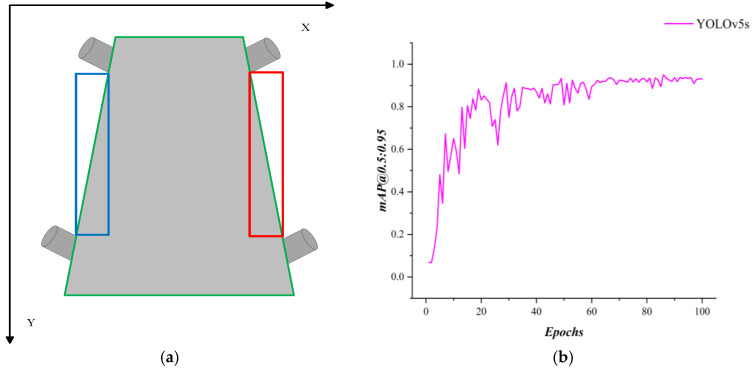
(**a**) Object detection with diagonal alignment of bounding boxes for belt edge detection, (**b**) Training curve of YOLOv5s. Conveyor belt edge detection based on object detection.

In some studies, researchers have employed object detection models to achieve belt deviation detection by predicting rectangular bounding boxes of the conveyor belt region and aligning their diagonals with the belt edges. As shown in Figure 21, the basic principle of the proposed method is illustrated together with the training curve of YOLOv5s for mAP@0.5:0.95 in this scenario.

**Figure 22:**
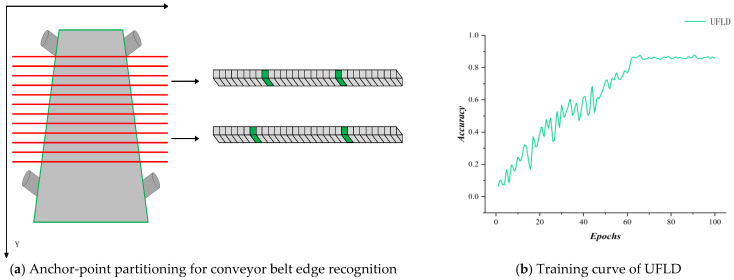
Principle illustration of fast lane detection (UFLD) and its training curve in the present scenario.

Another category of methods is based on anchor-point partitioning, such as UFLD, which locates conveyor belt edges through a set of predefined anchor points. The figure below illustrates the principle of this method, along with its accuracy curve in the present scenario. As shown, the UFLD method reaches an accuracy of approximately 0.85 after 100 epochs, but exhibits slow convergence and significant oscillations, indicating insufficient stability of this approach in the conveyor belt deviation detection task. As shown in Figure 22.

**Figure 23:**
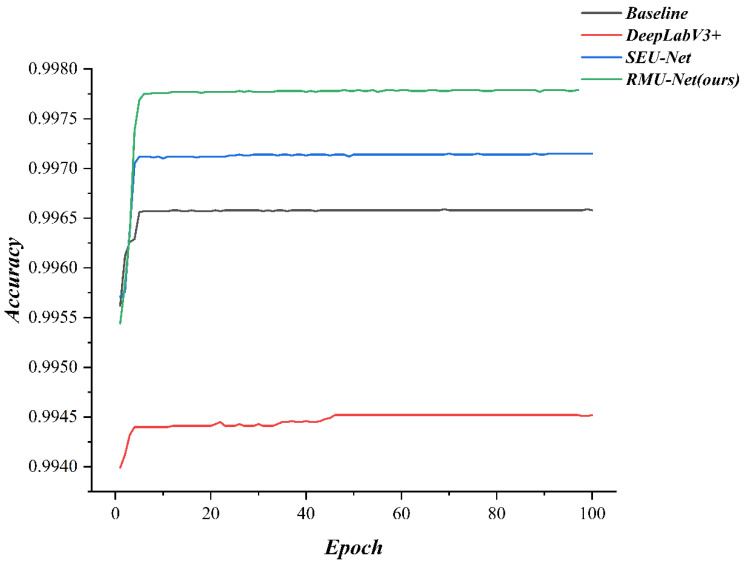
Accuracy curves of the proposed method and the baseline.

As shown in Figure 23, the accuracy curves of the proposed method, SEU-Net, and DeepLabV3+ are presented. Compared with YOLOv5s and UFLD, the proposed approach not only achieves a higher final accuracy of 99.77%—surpassing the baseline U-Net (99.65%) and significantly outperforming detection-based methods—but also demonstrates superior convergence stability and boundary representation accuracy.

### 3.4. Visualization Analysis

This section presents the recognition performance and robustness of the proposed method under different scenarios, and provides a visual comparison among five methods—the proposed method, YOLOv5s, UFLD, SEU-Net, and DeepLabV3+. The evaluation scenarios include normal illumination, strong light interference, rainy conditions, low-light environments, and severe belt deviation.

**Figure 24:**
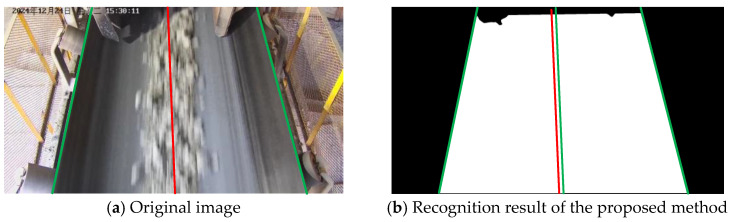

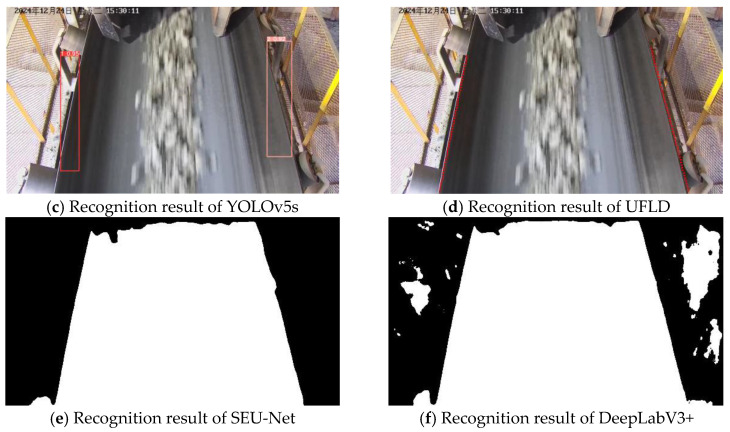
Recognition comparison under normal lighting conditions.

As illustrated in Figure 24, under normal lighting conditions, all methods are able to recognize conveyor belt edges to a reasonable extent. However, the results indicate that YOLOv5s, due to its reliance on the diagonal approximation of bounding boxes, exhibits noticeable boundary offsets; UFLD suffers from jitter during anchor-point fitting; SEU-Net exhibits limited capability in edge extraction and tends to produce adhesion in regions with similar color tones, while DeepLabV3+ generates a higher number of false detections in complex backgrounds. In contrast, the proposed method produces more complete, smoother, and more accurate edge delineations that align closely with the ground-truth boundaries.

**Figure 25:**
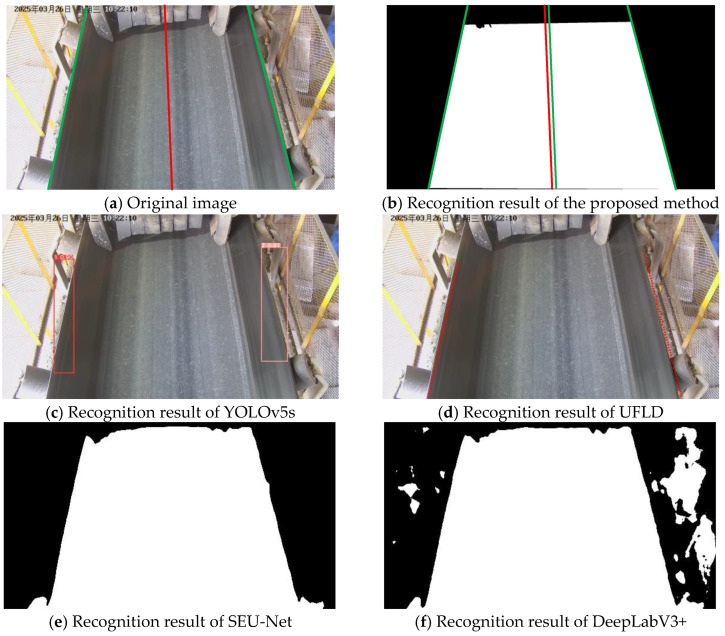
Recognition comparison under strong light conditions.

As shown in Figure 25, under strong light interference, partial regions exhibit overexposure. The bounding boxes generated by YOLOv5s are significantly affected, resulting in obvious boundary offsets. The UFLD method shows unstable anchor-point predictions, leading to edge curves that deviate from the true conveyor belt boundary. SEU-Net still exhibits adhesion between the roller and the belt regions, while DeepLabV3+ remains susceptible to background interference and continues to produce false detections in complex scenes. In contrast, the proposed method maintains stable edge detection performance.

**Figure 26:**
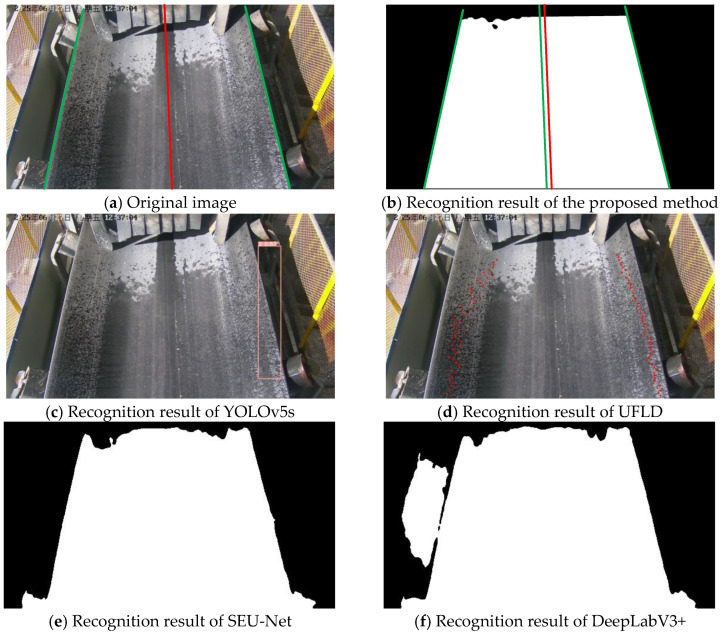
Recognition comparison under rainy conditions.

As illustrated in Figure 26, in rainy environments, raindrops and blurring effects make it difficult for conventional methods to extract edges reliably. YOLOv5s suffers from missing detection boxes and boundary shifts. UFLD fails to generate sufficient anchor points, which leads to inaccurate edge fitting. SEU-Net continues to exhibit adhesion between the roller and the conveyor belt regions, whereas DeepLabV3+ shows adhesion in areas where the background and the belt have similar color tones. By leveraging global context modeling in the segmentation framework, the proposed method effectively distinguishes between background and edge regions.

**Figure 27:**
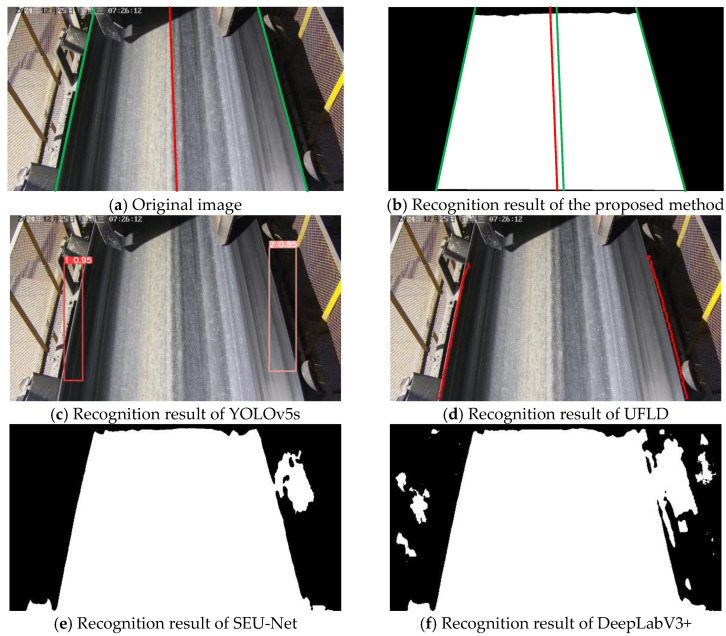
Recognition comparison under low-light conditions.

As shown in Figure 27, under low-light conditions, the right side of the conveyor belt exhibits insufficient brightness. YOLOv5s demonstrates imprecise boundary detection, and UFLD yields significantly shifted outputs. SEU-Net tends to produce adhesion in shadow-affected regions, whereas DeepLabV3+ exhibits similar adhesion problems and further misclassifies a considerable amount of background as target regions. Benefiting from the strong feature representation of deep layers, the proposed method is able to sustain stable detection accuracy and maintain edge continuity even under dim illumination.

**Figure 28:**
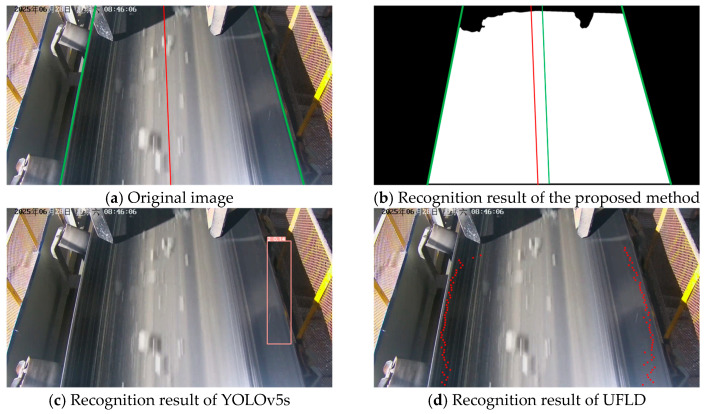

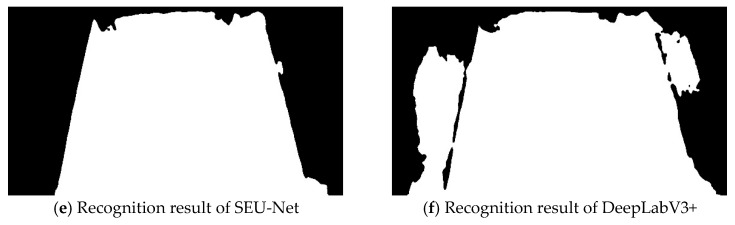
Recognition comparison under severe deviation conditions (the right roller is almost entirely occluded by the belt).

As illustrated in Figure 28, under severe deviation, the right roller is almost completely occluded by the conveyor belt. YOLOv5s continues to experience missing detection boxes with relatively low confidence levels, and UFLD presents noticeable deviations in anchor-point localization. SEU-Net still exhibits adhesion artifacts and imprecise edge detection of the conveyor belt, while DeepLabV3+ is strongly affected by background noise, leading to numerous false responses. The proposed method, however, successfully extracts edge information and produces boundary delineations that closely match the true belt edges, demonstrating its practicality and robustness.

From the visual comparisons across diverse environments, YOLOv5s demonstrates limited adaptability to complex industrial scenarios because of its dependence on rectangular bounding boxes, whereas UFLD suffers from degraded edge continuity under sparse anchor distributions or strong environmental interference. SEU-Net shows inadequate precision in segmenting edge details, leading to adhesion between the rollers and the conveyor belt, while DeepLabV3+ is prone to numerous false detections in cluttered or complex backgrounds. In contrast, the proposed method consistently delivers accurate and stable recognition across all conditions, and shows enhanced robustness and application potential in extreme cases such as strong light, rainfall, and severe belt deviation.

## 4. Conclusions

This study proposes a vision-based deep learning solution to address the practical engineering requirements of conveyor belt deviation detection. The effectiveness of the proposed method is validated through extensive experiments under multiple working conditions. The results demonstrate that the method can stably and accurately extract belt edges in complex environments and, by combining the constructed centerline reference, enables real-time identification of deviation states and quantitative measurement of displacement. The findings further verify the superior performance of segmentation-based approaches in this task. However, several limitations and possible improvements remain:

(1) Limited robustness to illumination changes and contamination. Although the model performs well under strong- and low-light conditions, severe dust or lens contamination may still degrade edge features, affecting the stability of deviation estimation.

(2) High model complexity. While the current network architecture ensures high accuracy, it incurs substantial computational costs and relatively low inference speed. Future work will consider lightweight network design or model pruning to improve real-time performance.

(3) Restricted experimental scope. The dataset used in this study was mainly constructed from a single mining site and does not cover extreme conditions such as rain, snow, or heavy occlusion. Expanding the dataset diversity will be important for improving generalization capability.

(4) Incomplete deployment optimization. Although the system can operate in real time on an industrial computer, further optimization for embedded edge devices is still needed. Future research will explore the integration of edge computing and multi-modal fusion to enhance the system’s stability and adaptability in practical deployments.

Overall, this study verifies the feasibility of applying deep learning techniques to conveyor belt deviation detection from a methodological perspective, while also highlighting innovation in both experimental design and engineering value. Future work will further explore lightweight model design, edge computing deployment, and multimodal information fusion to enhance real-time performance and strengthen applicability and deployment potential in industrial field environments.

## Figures and Tables

**Table 1:** Training Hyperparameters.

Training Metric	Value	Validation Metric	Value
Epochs	100	Betas	0.9, 0.999
Batch Size	4	Optimizer	AdamW
Weight Decay	0.0001	Learning Rate	0.0005

**Table 2:** Experimental Software and Hardware Configuration.

Component	Specification
Operating System	Windows 10 Professional
Experimental Framework	Python 3.9.21/PyTorch 2.7.1
CPU	14th Gen Intel(R) Core(TM) i9-14900
GPU	NVIDIA GeForce RTX 5070
RAM	32 G

**Table 3:** Ablation study results.

Baseline	ResNet	MASAG	Loss(CE + Dice)	Loss(CE + Dice + Boundary)	Accuracy(%)	IoU(%)
√			√		99.655	99.403
	√		√		99.758	99.516
		√	√		99.762	99.521
	√	√	√		99.768	99.530
	√	√		√	99.778	99.538

The check marks (“√”) in the table indicate that the corresponding component or setting is included in that experiment. For example, a check mark under “ResNet” means that the ResNet encoder was used in that configuration.

**Table 4:** Comparison of different methods in terms of Accuracy, Frame Rate, and Computational Complexity.

Model	Category	Accuracy (%)	FPS	GFLOPs
Baseline	Segmentation	99.655	20	226.80
DeepLabV3+	Segmentation	99.452	12	378.72
SEU-Net	Segmentation	99.715	18	252.62
YOLOv5s	Object Detection	93.105	56	81.36
UFLD	The Lane Detection	85.121	50	90.72
Ours	Segmentation	99.778	16	283.87

**Table 5:** Mean square error (MSE) between the detected centerline and the reference line under different environmental conditions.

Environmental Condition	Number of Images	MSE
Normal lighting conditions	1000	25.82
Strong light interference	500	26.71
Rainy environments	500	28.56
Low-light conditions	500	27.66

## Data Availability

The data that support the findings of this study are available on request from the corresponding author, upon reasonable request.
